# Supplementary material for: Meta-Analysis of European Clinical Trials Characterizing the Healthy-Adult Serum 25-hydroxyvitamin D Response to Vitamin D Supplementation
Source: Nutrients. 2023 Sep 14;15(18):3986. doi: 10.3390/nu15183986 (PMC10537880; doi:10.3390/nu15183986)
Supplement: Supplementary file 1 [file nutrients-15-03986-s001.zip › nutrients-2609950-supplementary.pdf]

1. Supplementary Materials

File S1. Pubmed literature search terms

#1 Search ("vitamin d"[MeSH Terms] OR "ergocalciferols"[MeSH Terms] OR vitamin d[Text Word] OR "calcifediol"[MeSH Terms] OR calcifediol[Text Word] OR "25-hydroxyvitamin D"[All Fields] OR 25 hydroxyvitamin d[Text Word] OR "cholecalciferol"[MeSH Terms] OR cholecalciferol[Text Word] OR "vitamin d deficiency"[MeSH Terms] OR vitamin d deficiency[Text Word])

#2 Search ("dietary supplements"[MeSH Terms] OR dietary supplement[Text Word])

#3 Search (#1 AND #2)

These results were then pre-filtered in PUBMED by any type of study type:

Clinical Study, Clinical Trial, Clinical Trial, Phase I, Clinical Trial, Phase II, Clinical Trial, Phase III, Clinical Trial, Phase IV, Comparative Study, Controlled Clinical Trial, Dataset, Evaluation Study, Multicenter Study, Observational Study, Pragmatic Clinical Trial, Randomized Controlled Trial, Research Support, American Recovery and Reinvestment Act, Research Support, N.I.H., Extramural, Research Support, N.I.H., Intramural, Research Support, Non-U.S. Gov't, Research Support, U.S. Gov't, Non-P.H.S., Research Support, U.S. Gov't, P.H.S., Research Support, U.S. Gov't, Twin Study, Validation Study.

Figure S1. Risk of bias and Level of evidence evaluation.

	Random sequence generation (selection bias)	Allocation concealment (selection bias)	Blinding of participants and personnel (performance bias)	Blinding of outcome assessment (detection bias)	Incomplete outcome data (attrition bias)	Selective reporting (reporting bias)	Other bias	Oxford 2011 Level of evidence
Agergaard 2015	+	?	+	+	+	?	+	2
Andersen 2008	+	+	+	?	+	?	+	3
Bischoff-Ferrari 2020	+	+	+	+	+	?	+	2
Bolton-Smith 2007	+	+	+	+	+	?	+	2
Brazier 2005	+	+	?	?	+	?	+	3
Cashman 2008	+	+	+	+	+	?	+	2
Cashman 2009	+	+	+	+	+	?	+	2
Cashman 2012	+	+	+	+	+	?	+	2
Cashman 2014	+	+	+	+	+	?	+	2
Cefalo 2018	+	+	+	+	+	?	+	3
Chel 2008	+	?	?	?	+	?	−	3
Close 2013	+	+	+	+	+	?	+	3
Goncalves-Mendes 2019	+	+	+	+	+	?	+	3
Grimnes 2011	+	+	+	+	+	?	+	2
He 2016	+	+	?	?	+	?	+	3
Heikkinen 1998	+	?	?	?	+	?	−	3
Holmlund-Suila 2016	?	+	?	?	+	?	+	3
Itkonen 2016	?	?	?	?	+	?	+	3
Jastrzebski 2016	?	+	?	?	+	?	+	3
Kashi 2021	+	+	+	+	+	?	+	3
Kasprowicz 2020	?	?	+	?	+	?	+	3
Kjaergaard 2012	+	+	+	?	+	?	+	2
Knutsen 2014	+	+	+	+	+	?	+	2
Kubiak 2018	+	+	+	+	+	?	+	2
Kujach 2020	−	+	?	?	?	?	−	3
Laaksi 2010	+	+	+	+	−	?	+	3

Lehmann 2015	+	+	+	+	+	?	+	2
Lerchbaum 2017	+	+	+	+	+	?	+	2
Lerchbaum 2019	+	+	+	+	+	?	+	2
Lithgow 2018	+	+	+	+	+	?	+	2
Maboshe 2021	+	+	+	+	+	?	+	2
Martineau 2015	+	+	+	+	+	?	+	2
Mielgo-Ayuso 2018	+	+	+	+	+	?	+	2
Nygaard 2014	+	+	+	−	+	?	+	2
O'Sullivan 2011	+	+	+	+	+	?	+	2
Ooms 1995	+	+	+	+	+	?	+	3
Osmancevic 2016	?	+	?	?	−	?	+	3
Prietl 2014	+	+	+	+	+	?	+	2
Sneve 2008	?	+	+	+	+	?	+	3
Trummer 2020	+	+	+	+	+	?	+	2
Urbain 2011	+	+	−	−	+	?	+	3
Vaes 2018	+	+	+	+	+	?	+	2
Välimäki 2016	?	?	?	?	?	?	+	3
Viljakainen 2009	?	?	?	?	+	?	+	3
Wamberg 2013	+	+	+	+	+	?	+	3
Wood 2012	+	+	+	+	+	?	+	2
Wyon 2016	+	+	+	+	+	?	+	2
Wyon 2021	+	+	+	+	+	?	+	2
Zittermann 2009	+	+	+	+	+	?	+	2

Figure S2. Funnel plot of the overall population.

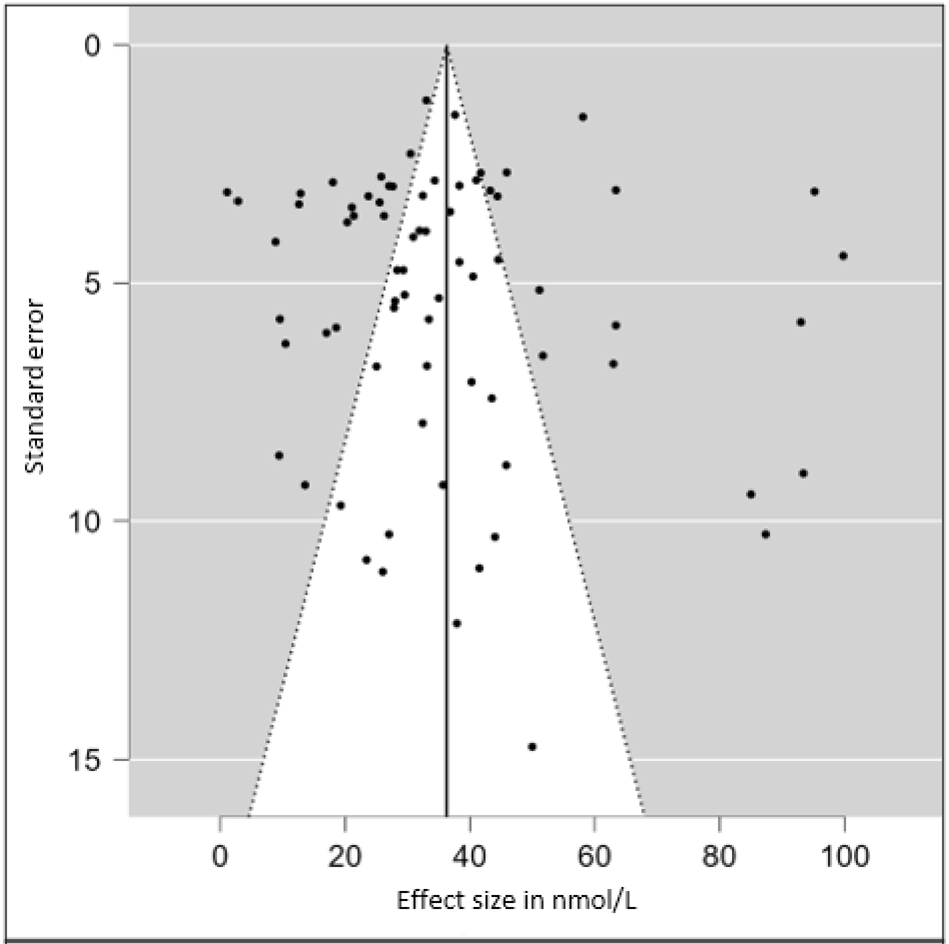

Regression test for funnel plot - asymmetry ("Egger's Test")

	z	p
sei	1.314	0.189

Figure S3. Forest Plot Northern latitude <50 nmol/L ethnic minorities vs. northern European population in 18-59 year olds.

Study or Subgroup	Mean Difference	SE	Placebo Total	Vitamin D Total	Weight	Mean Difference IV, Random, 95% CI
**1.43.1 18-59_Northern Latitudes <50nmol/L_Ethnic Minorities**						
Andersen 2008 (Men 10 µg)	21.3435	3.5863	19	10	5.4%	21.34 [14.31, 28.37]
Andersen 2008 (Men 20 µg)	36.8212	3.4989	26	10	5.4%	36.82 [29.96, 43.68]
Andersen 2008 (Women 10 µg)	30.4437	2.2816	21	9	5.5%	30.44 [25.97, 34.92]
Andersen 2008 (Women 20 µg)	34.3428	2.8456	22	10	5.4%	34.34 [28.77, 39.92]
Knutsen 2014 (10 µg)	18	2.8814	69	35	5.4%	18.00 [12.35, 23.65]
Knutsen 2014 (25 µg)	27	2.9601	84	36	5.4%	27.00 [21.20, 32.80]
Osmancevic 2016 (20 µg)	13.5267	9.2438	13	5	4.8%	13.53 [-4.59, 31.64]
Osmancevic 2016 (40 µg)	35.663	9.2418	12	4	4.8%	35.66 [17.55, 53.78]
**Subtotal (95% CI)**			**266**	**119**	**42.2%**	**27.62 [22.35, 32.89]**
Heterogeneity: Tau² = 39.91; Chi² = 31.21, df = 7 (P < 0.0001); I² = 78%						
Test for overall effect: Z = 10.27 (P < 0.00001)						
**1.43.2 18-59_Northern Latitude <50 nmol/L_Northern population**						
Agergaard 2015 (young)	23.7	3.1728	7	10	5.4%	23.70 [17.48, 29.92]
Grimnes 2011	99.8	4.4284	49	45	5.3%	99.80 [91.12, 108.48]
Heikkinen 1998	12.8	3.116	17	18	5.4%	12.80 [6.69, 18.91]
Holmlund-Suila 2016 (Obese)	26	11.0633	7	9	4.6%	26.00 [4.32, 47.68]
Kashi 2021	8.8	4.1332	32	29	5.4%	8.80 [0.70, 16.90]
Kjaergaard 2012	95.2	3.076	120	110	5.4%	95.20 [89.17, 101.23]
Kubiak 2018	58.1	1.5076	208	203	5.5%	58.10 [55.15, 61.05]
Lithgow 2018	40.25	7.0755	10	10	5.1%	40.25 [26.38, 54.12]
Maboshe 2021	35	5.3151	26	29	5.3%	35.00 [24.58, 45.42]
Nygaard 2014	51.14	5.1452	22	18	5.3%	51.14 [41.06, 61.22]
Wamberg 2013	63.4	5.8891	22	21	5.2%	63.40 [51.86, 74.94]
**Subtotal (95% CI)**			**520**	**502**	**57.8%**	**46.94 [28.74, 65.14]**
Heterogeneity: Tau² = 919.63; Chi² = 699.27, df = 10 (P < 0.00001); I² = 99%						
Test for overall effect: Z = 5.05 (P < 0.00001)						
**Total (95% CI)**			**786**	**621**	**100.0%**	**38.67 [27.25, 50.08]**
Heterogeneity: Tau² = 617.15; Chi² = 962.31, df = 18 (P < 0.00001); I² = 98%						
Test for overall effect: Z = 6.64 (P < 0.00001)						
Test for subgroup differences: Chi² = 3.99, df = 1 (P = 0.05), I² = 75.0%						

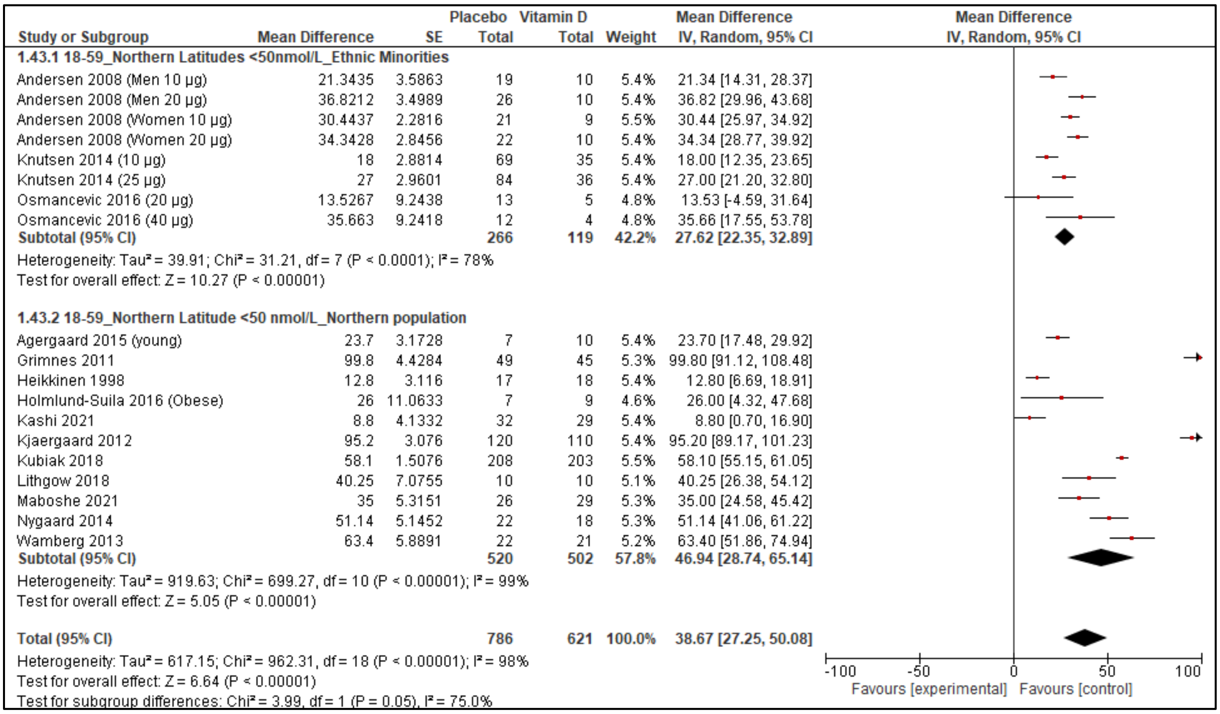

Table S1. Subgroup characteristics +/- calcium addition.

Subgroup (number of study arms)	Baseline 25(OH)D in nmol/L*	Dose µg/d*	Weighted mean difference in nmol/L	Serum increment in nmol/L per 2,5 µg/d Vit.D[ǂ]
Calcium addition (n=11)	50.67	41.91	31.70	1.68
No Calcium addition (n=62)	38.90	44.93	37.17	1.73
Calcium addition <50 nmol/L (n=3)	29.19	21.80	27.51	3.72
No Calcium addition <50 nmol/L (n=38)	32.54	64.68	39.52	1.62
Calcium addition ≥50 nmol/L (n=8)	58.90	50.00	33.31	1.34
No Calcium addition ≥50 nmol/L (24)	62.00	56.39	32.34	1.05
Calcium addition 18-59 Jahre (n=6)	47.28	51.82	34.90	1.50
No Calcium addition 18-59 Jahre (n=48)	44.78	72.24	37.69	1.23
Calcium addition ≥60 Jahre (n=6)	53.68	28.03	27.80	2.31
No Calcium addition ≥60 Jahre (n=14)	38.87	28.02	35.80	3.18

*: Weighted mean is the product of percentage weighting of studies and corresponding parameter

ǂ Increment 25(OH)D in nmol/L per 2.5µg/d (100 IU/d) was calculated as follows: [(Achieved weighted mean 25(OH)D concentration - Baseline weighted mean 25(OH)D concentration)/vitamin D dose µg/d)] × 2,5
